# Redox Homeostasis in Thyroid Cancer: Implications in Na^+^/I^−^ Symporter (NIS) Regulation

**DOI:** 10.3390/ijms23116129

**Published:** 2022-05-30

**Authors:** Juliana Cazarin, Corinne Dupuy, Denise Pires de Carvalho

**Affiliations:** 1 Instituto de Biofísica Carlos Chagas Filho, Universidade Federal do Rio de Janeiro, Rio de Janeiro 21941-902, Brazil; 2 UMR 9019 CNRS, Université Paris-Saclay, Gustave Roussy, 94800 Villejuif, France; corinne.dupuy@gustaveroussy.fr

**Keywords:** thyroid cancer, radioiodine therapy, NIS, reactive oxygen species

## Abstract

Radioiodine therapy (RAI) is a standard and effective therapeutic approach for differentiated thyroid cancers (DTCs) based on the unique capacity for iodide uptake and accumulation of the thyroid gland through the Na^+^/I^−^ symporter (NIS). However, around 5–15% of DTC patients may become refractory to radioiodine, which is associated with a worse prognosis. The loss of RAI avidity due to thyroid cancers is attributed to cell dedifferentiation, resulting in NIS repression by transcriptional and post-transcriptional mechanisms. Targeting the signaling pathways potentially involved in this process to induce de novo iodide uptake in refractory tumors is the rationale of “redifferentiation strategies”. Oxidative stress (OS) results from the imbalance between ROS production and depuration that favors a pro-oxidative environment, resulting from increased ROS production, decreased antioxidant defenses, or both. NIS expression and function are regulated by the cellular redox state in cancer and non-cancer contexts. In addition, OS has been implicated in thyroid tumorigenesis and thyroid cancer cell dedifferentiation. Here, we review the main aspects of redox homeostasis in thyrocytes and discuss potential ROS-dependent mechanisms involved in NIS repression in thyroid cancer.

## 1. Introduction

The unique ability of the thyroid gland to accumulate iodide (I^−^) is the basis of radioiodine (RAI) therapy, which has been used for decades as an effective therapy for differentiated thyroid cancer (DTC) treatment [1,2]. However, around 5–15% of DTCs become radioiodine-refractory (RAIR), which is associated with a worse prognosis [3,4,5]. Thus, managing RAIR metastatic thyroid cancer is challenging, and must include alternative therapeutic approaches. Although tyrosine kinase inhibitors (TKIs) bring significant therapeutic benefit to patients with RAI-refractory metastatic thyroid cancer, drug resistance and adverse effects that compromise patient quality of life may limit treatment responses [6,7]. Alternative potential tools include restoration of the radioiodine sensitivity of those tumors, which is the basis of redifferentiation strategies under current investigation [8].

Under physiological conditions, the thyroid gland can accumulate iodide in concentrations up to 40 times greater than those of plasma, which is attributed to a very specialized and tissue-specific iodine-handling machinery [9]. The Na^+^/I^−^ symporter (NIS), located in the basolateral membrane of thyrocytes, mediates active iodide uptake from the bloodstream to the intracellular compartment using the Na^+^ gradient generated by the Na^+^/K^+^ ATPase and the membrane potential as driving forces [10,11]. Additionally, the KCNQ1-KCNE2 K^+^ channel, also located in the basolateral membrane, has been shown to be required for NIS-mediated I^−^ uptake in thyroid tissue [12,13]. In the apical membrane, the iodide is oxidized to iodine and covalently incorporated into thyroglobulin (TG) in a reaction catalyzed by thyroperoxidase (TPO) [14]. This iodide organification process is essential for hormonal biosynthesis and prolongs iodide retention in the thyroid gland, which improves the tumor-absorbed dose of radiation and consequently the cytotoxic efficacy of radioiodine [15,16]. Thus, RAI therapy’s effectiveness is greatly dependent on proper NIS function but is also influenced by other specific factors of thyroid physiology.

Loss of RAI avidity in thyroid cancer is attributed to thyrocyte dedifferentiation, which results in the decreased expression of iodine-handling machinery genes including the NIS [17]. In corroboration, less-differentiated thyroid cancers, including poorly differentiated carcinomas (PDTC) and anaplastic carcinomas, are usually bad responders to RAI [18]. In addition to the repression of NIS expression, increased NIS internalization from the basolateral membrane has been implicated in the inability of thyroid cancers to respond to radioiodine [19,20]. Thus far, NIS impairment in thyroid cancers has not been attributed to mutations in the NIS gene, suggesting that transcriptional and post-transcriptional mechanisms are mainly involved. Therefore, targeting the signaling pathways involved in NIS repression might promote thyroid redifferentiation of RAI-refractory tumors, allowing de novo iodide uptake and effective radioiodine treatment.

MAPK pathway inhibitors have been shown to re-induce iodide uptake in RAI-refractory thyroid tumors in some patients, but not in others [21,22], suggesting that simultaneous or compensatory mechanisms might be involved in NIS regulation. Other signaling pathways, including Smad signaling, PI3K/mTOR, Notch, and the β-catenin pathway, have also been implicated in NIS repression in thyroid cancer, and might be potential targets for “redifferentiation” strategies [23]. Emerging evidence shows that redox imbalance is involved in thyroid tumorigenesis and dedifferentiation [24,25,26]. This review addresses how reactive oxygen species (ROS) impact NIS function in cancer and non-cancer contexts, contextualizing potential redox-related mechanisms implicated in NIS repression in thyroid cancer.

## 2. Redox Homeostasis in the Thyroid Gland

Reactive oxygen species (ROS) comprise a large group of highly reactive molecules derived from O_2_ reduction, which includes radical species such as superoxide (O_2_^•−^), hydroxyl (OH^•^), and peroxyl (RO_2_^•^), and non-radical species such as singlet oxygen (^1^O_2_), hypochlorous acid (HOCl), and the most biologically relevant, hydrogen peroxide (H_2_O_2_) [27]. Cellular ROS are produced by multiple sources, including mitochondrial electron-transport chain, nitric oxide synthase (eNOS), P450 enzymes, cyclooxygenase, and lipoxygenase as a by-product of metabolism. In contrast, NADPH oxidases produce ROS as their exclusive function. ROS levels result from a balance between their production and disposal by enzymatic (catalase, superoxide dismutase, glutathione peroxidase, thioredoxin reductase, and peroxiredoxins) and non-enzymatic (glutathione, β-carotene, uric, acid, vitamin C and E) cell antioxidant systems [28]. ROS react with proteins, nucleic acids, lipids, and inorganic molecules, inducing reversible or non-reversible modifications that can impact molecule structure and function, acting on multiple physiological and pathophysiological processes [29].

Physiologically, thyroid cells produce H_2_O_2_ within the follicular lumen during hormonal synthesis. H_2_O_2_ is required for the TPO-mediated oxidative iodination of tyrosine residues of thyroglobulin (TG), which will further allow T3 and T4 synthesis [30]. Dual-oxidase 2 (DUOX2), a member of the NADPH oxidase family, localized on the apical membrane of thyrocytes, is the source of H_2_O_2_ required for hormonal biosynthesis [31,32,33,34]. Indeed, loss-of-function mutations in DUOX2 or its maturation factor, DUOXA2, have been found in patients with congenital hypothyroidism and induced dyshormonogenesis in mouse models [33,35,36,37].

The amount of H_2_O_2_ produced by thyrocytes is quantitatively significant, being comparable to that produced by activated macrophages [38,39]. However, whereas macrophages are short-lived, the life of adult thyrocytes is around seven years, suggesting that adaptive mechanisms might prevent the deleterious effects of ROS exposure [40]. Different aspects of H_2_O_2_ metabolism have been proposed to protect thyroid cells from toxicity: (1) DUOX2-mediated H_2_O_2_ production is tightly regulated and restricted to the apical membrane–luminal interface where it is consumed and degraded by TPO; (2) the thyrocyte apical membrane exhibits poor permeability to H_2_O_2_; and (3) refined intracellular H_2_O_2_ detoxifying mechanisms exist [39,41].

Indeed, thyrocytes are more resistant to the cytotoxic effects of H_2_O_2_ than T cells, due to the activation of transcriptional responses that increase antioxidant defenses, especially glutathione peroxidase (GPx) [42]. In addition to GPx, the increased expression of other antioxidant enzymes, such as thioredoxin reductase (TrxR) and peroxiredoxins (Prx), has been reported during thyroid hormone synthesis and might be implicated in the regulation of redox homeostasis in physiological conditions [43,44]. The nuclear factor erythroid 2-related factor 2 (Nrf2) is a key regulator of the transcription of antioxidant enzymes. In thyrocytes, it positively regulates Gpx2 and Txnrd1, preventing intrathyroidal oxidative damage in response to iodide-induced ROS [45].

Oxidative stress (OS) results from the imbalance between ROS production and depuration that favors a pro-oxidative environment, which can result from increased ROS production, decreased antioxidant defenses, or both [46]. OS supports multiple stages of tumorigenesis by inducing oxidative DNA damage and genomic instability, sustaining proliferative pathways and cell survival, and promoting angiogenesis and metastasis [47]. Indeed, cancer cells usually exhibit increased ROS levels because of multiple stimuli, including hypoxia, metabolic imbalance, oncogene activation, and endoplasmic reticulum (ER) stress [29]. Thyroid cancer tissue exhibits increased ROS levels compared with normal thyroids [26,48], and a pro-oxidant environment has been implicated in chromosomal aberrations and the dedifferentiation of thyroid cancer cells [24,25,26,48]. 

**Figure 1:**
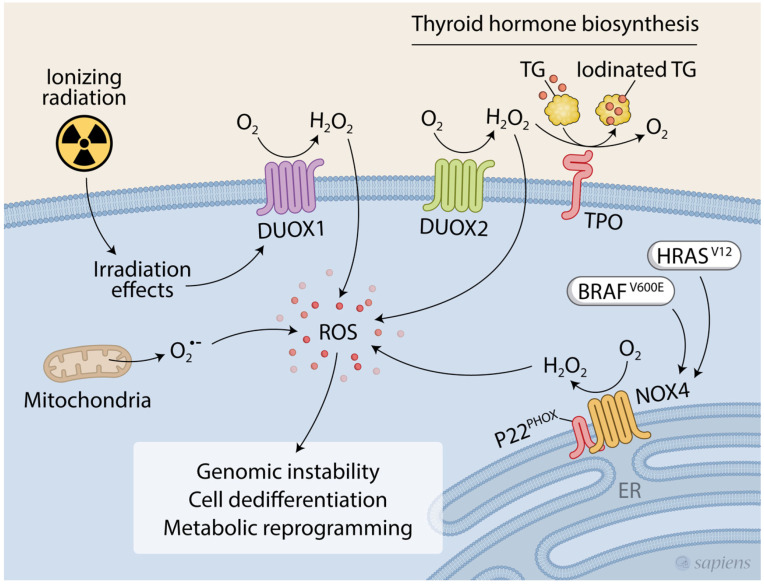
The role of oxidative stress in thyroid carcinogenesis. A pro-oxidant environment is associated with thyroid tumorigenesis, and NADPH oxidases have been described as important ROS sources. Ionizing radiation, a risk factor for thyroid cancer, induces DUOX1-dependent H_2_O_2_ production, resulting in DNA damage and potentially genomic instability. NOX4 is upregulated in PTCs and is positively regulated by the oncogenes BRAF^V600E^ and HRAS^V12^. Increased NOX4 has been implicated in thyroid cancer dedifferentiation and genomic instability. DUOX2 is the source of H_2_O_2_ for thyroid hormone biosynthesis in the apical membrane of thyrocytes, but its role in thyroid carcinogenesis is unclear.

NADPH oxidases are an important ROS source in PTCs [24,49]. As discussed previously, DUOX2’s central role in hormonal biosynthesis in the thyroid gland has been well-described. Across thyroid tumors, DUOX2 expression is widely variable, and its implication in thyroid carcinogenesis is not clear [46]. In addition to DUOX2, thyroid cells express two other ROS-generating enzymes from the NADPH oxidase family: DUOX1 and NOX4 [31,49]. Although their physiological roles are unknown, both are potentially involved in thyroid tumorigenesis [24,25,49,50,51] (Figure 1).

Ionizing radiation (IR) is a well-established risk factor for thyroid cancer in young people. It is significantly associated with the occurrence of cancer-driver RET/PTC translocation in vivo [52] and in vitro [53]. Interestingly, in vitro studies have revealed ROS as a mediator of this radiation-related effect, because the irradiation of cultured cells in the presence of antioxidants inhibited the occurrence of RET/PTC translocation [53]. Moreover, it has been shown that ionizing radiation induces the upregulation of DUOX1 expression and activity in thyroid cells in a p38 MAPK-dependent fashion, creating a persistent oxidative environment that could hypothetically promote tumorigenesis [24] (Figure 1). In corroboration, increased DUOX1 mRNA expression was found in radio-induced thyroid tumors [24].

NOX4 is expressed in various human and murine tissues, including the kidneys, lungs, heart, liver, vascular tissue, and thyroid gland [49,54]. NOX4 generates H_2_O_2_ and/or O_2_^•−^ in a constitutively active manner in intracellular compartments, including the endoplasmic reticulum, mitochondria, nucleus, and focal adhesions [55,56,57,58], requiring interaction with p22^phox^ for proper maturation and stabilization [59]. NOX4 staining was intracytoplasmic in human thyroid cells, whereas in rat thyrocytes, NOX4 was also detected in the plasma membrane [49,60].

NOX4 is upregulated in several types of human cancers, such as melanoma, ovarian, prostate, colorectal, and bladder cancer. It has been implicated in multiple aspects of tumorigenesis, including cell proliferation, migration, invasion, epithelial-to-mesenchymal transition (EMT), and metabolic rewiring [61,62,63,64,65,66]. NOX4 knockdown decreased tumor growth in a thyroid cancer xenograft mouse model, demonstrating the functional implications of NOX4 in thyroid tumorigenesis [67]. Indeed, both NOX4 and p22^phox^ are overexpressed in papillary thyroid cancers (PTCs), reinforcing the idea that thyroid cancer cells are under oxidative stress [49]. In human thyroid cells, the oncogene H-RasV12 increases NOX4 expression, resulting in DNA damage caused by increased ROS levels in the nuclear/perinuclear compartment, showing that NOX4 might impact genomic stability [51]. Additionally, NOX4-derived ROS are implicated in cell dedifferentiation and NIS repression in BRAF^V600E^-driven PTC, as discussed later [25] (Figure 1). Corroborating these findings, it has been demonstrated that the knockdown of NOX4 in the normal rat thyroid cell line FRTL-5 increases the mRNA expression of thyroid-related genes, including TTF2, TPO, and PAX8 [68].

Mitochondria are one of the main sources of intracellular ROS, and generate superoxide through complexes I and III as a by-product of oxidative phosphorylation [69]. The relationship between mitochondrial ROS and carcinogenesis in thyroid cancers is still poorly understood. Thyroid oncocytic tumors are characterized by the aberrant accumulation of enlarged and dysfunctional mitochondria with higher levels of mitochondrial DNA mutations [58,70]. These cells exhibit increased ROS levels correlated with the decreased activity of complexes I and III [71]. Clinically, oncocytic tumors are less responsive to radio-iodine therapy [72], and in PTCs, the oncocytic phenotype is significantly associated with the presence of BRAF mutations [73]. However, it is unknown whether the repressed ability to trap iodine by these tumors is functionally related to a pro-oxidant environment.

It has previously been demonstrated that NOX4 can be localized in the mitochondria, being a source of ROS within this compartment [56,74]. Mitochondrial NOX4 is activated when mitochondrial ATP levels are low, and induces drug resistance through ROS-dependent mechanisms in renal carcinoma cells [74]. NOX4 is required for mitochondrial ROS production in thyroid cancer cells under hypoxic conditions [67]. NOX4 or p22phox downregulation decreases mitochondrial ROS in hypoxic thyroid cancer cells, impairs HIF1α stabilization, HIF1α-induced glycolysis increment, and cell proliferation [67]. It is not clear whether this role of NOX4 is explicitly mediated by a pool of NOX4 located in mitochondria, because NOX4 knockdown is not mitochondria-directed.

Although a moderate increase in ROS fuels cancer initiation and progression, excessive ROS results in extensive macromolecular damage and cell toxicity [29]. Thus, fine-tuning ROS levels in an already pro-oxidant environment might be critical for cancer cell survival. It has been demonstrated that the PIM-1 kinase increases the protein expression of the antioxidants GPX1 and SOD2 in thyroid cancer cells, whereas its inactivation increases ROS [75]. Interestingly, the increased expression of GPX1 and SOD2 and augmented GPX and SOD activities have been reported in thyroid cancer when compared with normal tissue [48,75,76,77]. The expression of PIM-1 is overexpressed in PTCs and positively correlated with NOX4, GPX1, and SOD2 expression [75]. Interestingly, PIM-1, GPX1, and SOD2 protein expression are positively regulated by NOX4 in vitro, suggesting that NOX4 induces compensatory antioxidant responses through PIM-1 [75]. It is tempting to speculate that these compensatory antioxidant responses might be essential to maintain elevated ROS in concentration ranges compatible with thyroid cancer cell survival.

## 3. Evidence of NIS Regulation by ROS

Accumulating evidence suggests that NIS expression and function are regulated by ROS-dependent mechanisms in cancer and non-cancer contexts, such as during iodide overload. High iodide levels (I^−^) induce a transient inhibition of thyroid hormone biosynthesis, which is restored around two days after I^−^ administration, a mechanism of thyroid autoregulation known as the Wolff–Chaikoff effect [78]. Thyroid escape from the iodide inhibitory effect is attributed to reduced NIS iodide uptake and increased apical iodide efflux, which reduces concentrations of intracellular iodide and relieves thyroid function inhibition [78,79]. NIS-related responses to iodide overload are associated with dynamic changes in the cellular redox state [38,80,81,82].

Leoni and collaborators showed that I^−^ overload increased ROS levels and induced time-dependent decreases in NIS mRNA, protein, and activity in vitro and in vivo [80]. NIS recovery after I^−^ treatment depended on a compensatory increase in thioredoxin reductase antioxidant activity, showing that ROS levels are directly implicated in NIS regulation. In agreement, subsequent studies also found an ROS-dependent acute decrease in NIS mRNA and NIS inactivation at the plasma membrane in response to excess I^−^, which was reversed by ROS scavengers [83,84]. In addition to iodide overload, conditions that increase ROS levels in thyroid cells, including treatment with the endocrine disruptor bisphenol A, induced ROS-mediated NIS repression [85].

The mechanisms by which I^−^ increases ROS are still under investigation. Iodide has been shown to induce an acute increase in mitochondrial superoxide anions (O_2_^•−^), which induces PI3K/AKT pathway activation and NIS repression [83]. Recent data revealed NOX4 as another potential ROS source positively regulated by excess iodide [68]. Oglio et al. showed that I^−^ treatment increased NOX4 expression and induced ROS production, which was eliminated in the presence of the unspecific NADPH oxidase inhibitor DPI or siRNA against NOX4 in the rat thyroid cell line FRTL-5. NOX4 silencing inhibited iodide-induced *NIS* mRNA repression, indicating a central role of this NADPH oxidase in thyroid auto-regulation mediated by iodide [68].

NOX4 is a critical mediator of cell dedifferentiation and NIS repression in thyroid cancers harboring BRAF^V600E^ oncoprotein [25]. BRAF^V600E^ results from a gain-of-function transversion mutation in exon 15 (*BRAF* c.1799T>A). It is the most frequent genomic alteration found in PTCs, present in around 40–60% of cases, followed by RAS mutations (15%) and RET/PTC translocations (10–15%). Those genomic alterations are mutually exclusive and lead to constitutive activation of the MEK–ERK signaling pathway [86]. Clinically, the occurrence of BRAF^V600E^ has been associated with increased tumor size, disease recurrence, and mortality. However, there is no consensus on the independent prognostic value of this mutation in thyroid cancers [87,88,89,90].

It has extensively been demonstrated in vitro and in vivo that BRAF^V600E^ represses not only the expression of thyroid-specific genes involved in iodide-handling machinery, including *TPO*, *DUOX*, *TG*, and *NIS*, but also impairs NIS activity and targeting in the membrane [91,92]. Clinical studies also found a negative correlation between BRAF^V600E^ and NIS expression, and found that RAI-refractory metastatic thyroid cancer is enriched in BRAF mutations [93,94,95]. In agreement, high-throughput analysis of a large cohort of human PTC specimens also showed a negative correlation between the BRAF^V600E^ and thyroid differentiation score when compared with tumors harboring RAS mutations or RET/PTC translocations [86]. Overall, these data support the concept that BRAF^V600E^ is functionally involved in radioiodine resistance. However, it is worth mentioning that even though BRAF^V600E^ is a frequent mutation in DTCs, only a small subset of these tumors is radioiodine-resistant, suggesting that additional molecular events cooperate with BRAF^V600E^ mutation in the loss of RAI avidity.

Constitutive MAPK activation plays a central role in cell dedifferentiation in thyroid cancer. In PTCs, lower differentiation scores were associated with higher rates of MEK–ERK activation [86]. Interestingly, BRAF^V600E^-positive PTC exhibits strong activation of the ERK transcriptional program by escaping from ERK-mediated negative feedback loops [86,96]. Indeed, MEK–ERK pathway inhibition in BRAF^V600E^-induced thyroid cancer mouse models partially restores the expression of thyroid-related genes and iodide uptake [92,97]. These data motivated studies conducted with RAI-refractory thyroid cancer patients using MEK (selumetinib, trametinib, and cobimetinib) or BRAF^V600E^ inhibitors (dabrafenib or vemurafenib), or a combination of both as a redifferentiation strategy to improve RAI avidity and induce better responses to RAI therapy [18,21,22,98,99,100,101,102]. Patients harboring RAS mutations had a better response rate than patients carrying BRAF^V600E^ mutation, showing the development of more efficient MAPK inhibitors or a better comprehension of mechanisms that cooperate with BRAF^V600E^-mediated dedifferentiation is necessary for the design of new strategies to improve RAI responsiveness [21,101].

TGF-β1 has been described as a critical player in BRAF^V600E^-induced thyroid dedifferentiation [91]. In rat thyroid cells, Riesco-Eizaguirre and colleagues showed that BRAF^V600E^ increases TGF-β1 secretion, which acts through an autocrine-loop-activating SMAD pathway, inducing epithelial-to-mesenchymal transition (EMT) and cell migration, and repressing NIS expression and function [91]. In human PTC, TGF-β1 and components of the TGF-β cascade were found to be overexpressed, which was significantly correlated with BRAF status and reduced NIS expression in the cell membrane [91]. Azouzi et al. added a new piece to this puzzle, showing that NOX4 is upregulated by BRAF^V600E^–TGFB–SMAD3 signaling and induces ROS-mediated NIS downregulation in human thyroid cancer cells [25]. TCGA database analysis of human PTCs revealed that mRNA NOX4 levels are increased in tumors harboring BRAF^V600E^ as compared with wild-type BRAF [25]. Additionally, in BRAF^V600E^-positive PTCs, NOX4 expression was associated with increased ERK activation and was inversely correlated with NIS mRNA levels and thyroid differentiation scores [25].

Overall, these data suggest that ROS mediates NIS repression. However, the molecular mechanisms involved remain to be elucidated. Based on the current knowledge of NIS regulation, we will now discuss potential mechanisms involved in the redox regulation of NIS expression, activity, subcellular location, and protein stability.

### 3.1. Regulation of NIS Expression by ROS

PAX8 is a transcriptional factor required for thyroid development and differentiation that acts as a major regulator of *NIS* transcription. PAX8 binds to the *NIS* upstream enhancer (NUE) in human and rat thyrocytes and induces NIS expression [103,104]. As with other PAX family members, the PAX8 DNA binding activity depends on the redox state of two cysteine residues in its structure: Cys-45 and Cys-57. In thyroid cells, PAX8 binding to NUE and the induction of *NIS* transcription depend on PAX8 being converted to a reduced form by apurinic/apyrimidinic endonuclease 1 (APE1) and a reduction cascade involving thioredoxin reductase-1 (TxnRd1) [105,106]. These data agree with previous findings showing that increased thioredoxin reductase activity is associated with the recovery of NIS expression after iodide-mediated ROS increases in thyroid cells [80]. Thus, redox imbalance in thyroid cancer cells might reduce NIS expression by promoting Pax8 oxidation.

It is well-documented that NIS repression in thyroid cancer also involves epigenetic mechanisms. Hypermethylation of CpG islands in the *NIS* promoter has been found in thyroid tumors that harbor low levels of NIS expression and/or impaired iodide uptake [107,108]. In human thyrocytes, BRAF^V600E^ expression promotes hypermethylation of the *NIS* promoter and NIS repression, which is associated with increased levels of the DNA-methylating enzyme, DNA methyl-transferase 1 (DNMT1) [109]. Reduced histone acetylation in the *NIS* promoter has also been implicated in BRAF^V600E^-mediated NIS repression, corroborating the increment in NIS expression found in thyroid cancer cells treated with histone deacetylase inhibitors (HDACis) [110,111,112,113,114,115]. Finally, microRNAs (miRNAs) are also implicated in NIS regulation. miR-146b and miR-21 induce NIS repression and are inversely correlated with thyroid differentiation scores in PTCs [86,116,117].

Oxidative stress induces epigenetic alterations that support tumorigenesis by silencing tumor suppressor genes through the regulation of co-factor availability, miRNAs, and the epigenetic machinery involved in DNA methylation and histone modifications [118]. In colorectal cancer cells, H_2_O_2_ treatment increases both DNMT1 and HDAC1 expression and activity [119]. In addition, oxidative DNA damage induces the formation and relocation of silencing complexes containing DNMT1, contributing to the modifications of DNA methylation patterns seen in cancer cells [120]. Interestingly, miR-21, an oncogenic miRNA implicated in NIS regulation, is positively regulated by NADPH-oxidase-derived ROS in androgen-negative prostate cancer cells [121]. 

Finally, the subcellular site of ROS production and/or the spatial cellular distribution of antioxidant enzymes might be determinants for the mechanism elicited. NOX4, for example, produces H_2_O_2_ close to the nuclear compartment in thyroid cells [51], which might create a pro-oxidative microenvironment that enables the direct redox regulation of epigenetic events and transcription factors involved in NIS regulation [122]. Indeed, NOX4 was recently described as an essential mediator of hypoxia-induced histone methylation in pancreatic cancer cells [123].

### 3.2. Regulation of NIS Subcellular Location and Protein Stability by ROS

Loss of radioiodine avidity by thyroid cancers is caused by the repression of NIS expression, but also by its internalization from the basolateral plasma membrane to the intracellular compartment [19,20,91]. Although our knowledge of NIS trafficking regulation has significantly improved in the last few years, the mechanisms involved in this process, especially in cancer cells, remain unclear. NOX4 or p22^phox^ downregulation increased NIS plasma membrane expression in thyroid cancer cells, showing that NIS subcellular location might be a redox-regulated process [25]. 

The pituitary-tumor-transforming gene (PTTG)-binding factor (PBF) is upregulated in thyroid cancers, and it has been demonstrated to repress NIS function by decreasing NIS expression and inducing NIS endocytosis from the plasma membrane [124,125,126]. The PBF-mediated NIS internalization and repression of iodide uptake depends on PBF phosphorylation at residue Y174 by tyrosine kinase Src, which is abrogated by the Src inhibitor PP1 [127]. Src is a proto-oncogene tyrosine kinase activated by ROS [128]; therefore, it might be a pathway involved in NIS internalization mediated by oxidative stress. Interestingly, Src activation by NOX4-derived H_2_O_2_ has already been demonstrated in cancer and non-cancer contexts, suggesting a possible interplay between NOX4 and PBF signaling in thyroid cancer [129,130].

In follicular thyroid cancer (FTC) cells, it has been demonstrated that HIF1α-induced b-catenin activation induces the translocation of the NIS from the plasma membrane to the intracellular compartment. In a xenograft model, β-catenin knockdown increased the radioiodine treatment responsiveness of FTC cells that overexpressed HIF1α [131]. Interestingly, both HIF1α and β-catenin were found to be overexpressed in more aggressive thyroid cancer types and are redox-regulated in other cell types [132,133]. ROS trigger b-catenin signaling by oxidating the thioredoxin-like protein nucleoredoxin (NRX) and disrupting NRX–Dishevelled protein interaction, impairing β-catenin degradation [134]. At the same time, intracellular ROS activate HIF1α in both normoxia and hypoxia conditions [135]. In thyroid cancer cells, NOX4 has been shown to stabilize HIF1α in hypoxic conditions by increasing mitochondrial ROS, enabling cell proliferation [67]. Thus, HIF1α/β-catenin signaling might be a pathway potentially involved in the ROS-mediated regulation of the NIS in subcellular locations. 

In addition to NIS trafficking, the regulation of NIS protein stability impacts iodide uptake by thyrocytes. Our group previously demonstrated that AMPK-activated kinase (AMPK), a cellular energy sensor negatively regulated by TSH, induces NIS lysosomal degradation in rat thyroid cells [136]. Chai and colleagues recently demonstrated that high-mobility group box 1 (HMGB1) protein, a regulator of autophagy and chromatin remodeling, is upregulated in human thyroid cancer samples and represses iodide uptake by promoting NIS degradation [137]. HMGB1 induces autophagy and NIS lysosomal degradation by activating AMPK through an ROS-dependent mechanism [137], which corroborates previous findings showing that AMPK is redox-sensitive [138]. Interestingly, AMPK and its active form, p-AMPK, are upregulated in human PTCs [139].

### 3.3. Regulation of NIS Activity by ROS

Iodide-induced ROS has been shown to cause a rapid decrease in NIS activity, which is not related to protein internalization or preceded by alterations in mRNA or protein levels, which suggests an inactivation of membrane-bounded NIS by post-translational mechanisms [80,84]. In this context, ROS regulation of NIS activity might occur by both direct and indirect means. A potential direct mechanism relies on the oxidation of cysteine residues in NIS protein. Cysteine residues are sensitive to reversible redox modifications that impact protein conformation and function, both being identified as eligible ROS direct targets in the NIS structure [80]. However, no functional studies have been performed thus far to demonstrate the direct implication of these residues on NIS activity [80]. 

It was previously demonstrated that NIS activity is regulated by the phosphorylation of two serine residues in rat NIS protein: Ser-43 and Ser-581. The site-directed mutagenesis of these residues reduced the maximal iodide transport velocity by 40% and 60%, respectively [140]. The activities of a variety of serine/threonine kinases and phosphatases are redox-regulated; therefore, ROS might potentially inhibit NIS activity, indirectly, by modifying the NIS phosphorylation pattern [141].

## 4. Conclusions

**Figure 2:**
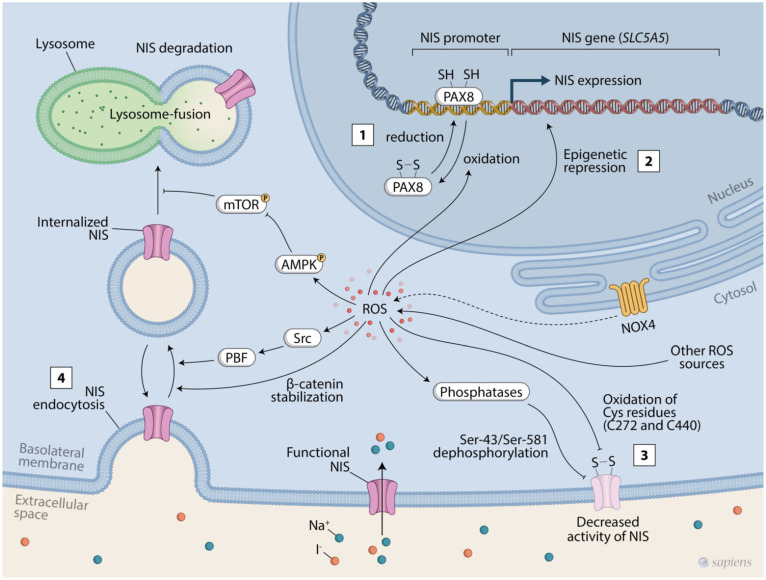
Mechanisms hypothetically involved in NIS redox regulation: (1) PAX8 oxidation results in reduced PAX8 DNA binding activity and the repression of *NIS* transcription; (2) ROS might mediate alterations of epigenetic events also promoting *NIS* transcriptional repression; (3) ROS might directly oxidize NIS protein or indirectly change the phosphorylation pattern of NIS protein, resulting in decreased NIS activity; (4) ROS might activate pathways involved in NIS endocytosis and autophagy, promoting NIS internalization and degradation.

ROS repress NIS expression and activity in cancer and non-cancer contexts at the transcriptional and post-transcriptional levels (Figure 2). Therefore, targeting redox homeostasis is a potential tool for promoting thyroid cancer cell redifferentiation. Although our understanding of thyroid redox homeostasis has evolved over the last decade, the molecular mechanisms involved in ROS-mediated NIS repression are not well-defined. Future studies are necessary to establish those mechanisms and evaluate whether they can be explored therapeutically to promote de novo iodine uptake in iodine-refractory thyroid tumors.
